# Assessment of the efficacy of antimalarial drugs recommended by the National Malaria Control Programme in Madagascar: Up-dated baseline data from randomized and multi-site clinical trials

**DOI:** 10.1186/1475-2875-7-55

**Published:** 2008-04-04

**Authors:** Didier Ménard, Arsène Ratsimbasoa, Milijaona Randrianarivelojosia, Léon-Paul Rabarijaona, Lucie Raharimalala, Olivier Domarle, Laurence Randrianasolo, Arthur Randriamanantena, Martial Jahevitra, Valérie Andriantsoanirina, Marie-Ange Rason, Rogelin Raherinjafy, Emma Rakotomalala, Luciano Tuseo, Andrianirina Raveloson

**Affiliations:** 1 Malaria Unit Research, Institut Pasteur de Madagascar, Antananarivo, Madagascar; 2 Epidemiology Unit, Institut Pasteur de Madagascar, Antananarivo, Madagascar; 3 Immunology Unit, Institut Pasteur de Madagascar, Antananarivo, Madagascar; 4 WHO Office of Madagascar and La Réunion, Antananarivo, Madagascar; 5 National Malaria Control Programme, Ministry of Health, Antananarivo, Madagascar

## Abstract

**Background:**

In order to improve the monitoring of the antimalarial drug resistance in Madagascar, a new national network based on eight sentinel sites was set up. In 2006/2007, a multi-site randomized clinical trial was designed to assess the therapeutic efficacy of chloroquine (CQ), sulphadoxine-pyrimethamine (SP), amodiaquine (AQ) and artesunate plus amodiaquine combination (ASAQ), the antimalarial therapies recommended by the National Malaria Control Programme (NMCP).

**Methods:**

Children between six months and 15 years of age, with uncomplicated falciparum malaria, were enrolled. Primary endpoints were the day-14 and day-28 risks of parasitological failure, either unadjusted or adjusted by genotyping. Risks of clinical and parasitological treatment failure after adjustment by genotyping were estimated using Kaplan-Meier survival analysis. Secondary outcomes included fever clearance, parasite clearance, change in haemoglobin levels between Day 0 and the last day of follow-up, and the incidence of adverse events.

**Results:**

A total of 1,347 of 1,434 patients (93.9%) completed treatment and follow-up to day 28. All treatment regimens, except for the chloroquine (CQ) treatment group, resulted in clinical cure rates above 97.6% by day-14 and 96.7% by day-28 (adjusted by genotyping). Parasite and fever clearance was more rapid with artesunate plus amodiaquine, but the extent of haematological recovery on day-28 did not differ significantly between the four groups. No severe side-effects were observed during the follow-up period.

**Conclusion:**

These findings (i) constitute an up-dated baseline data on the efficacy of antimalarial drugs recommended by the NMCP, (ii) show that antimalarial drug resistance remains low in Madagascar, except for CQ, compared to the bordering countries in the Indian Ocean region such as the Comoros Archipelago and (iii) support the current policy of ASAQ as the first-line treatment in uncomplicated falciparum malaria.

## Background

Despite major efforts made by national and international health organizations, malaria remains the most widespread infectious parasitic diseases and one of the most serious global health problems in the world [1]. In sub-Saharan African, the main problem is *Plasmodium falciparum *drug-resistance, especially with the spread of the parasite resistance to the inexpensive and widely used drugs, such as chloroquine (CQ) or sulphadoxine-pyrimethamine (SP) [2,3], and the major consequence is the use of ineffective antimalarial drugs leading to the increasing malarial incidence and mortality [4,5].

Therefore, substantial efforts have been made to encourage the monitoring and the evaluation of the antimalarial drugs resistance in the endemic countries for assessing regularly their antimalarial drug policies and ensuring a continued coverage of effective antimalarial treatment [6].

In Madagascar, since 2005, antimalarial treatment is in transition with the elaboration and the implementation of the new national policy for the fight against malaria [7]. The main modifications in term of drugs use has been the withdrawal of CQ in favour of artemisinin combination therapies (ACTs), as first-line (artesunate plus amodiaquine combination, ASAQ) and second-line treatment (artemether plus lumefantrine combination), and the use of the SP for intermittent preventive treatment for pregnant women (IPTp). This choice was guided by the recommendations of WHO and unpublished data from a clinical trial conducted on the island of Sainte Marie in 2004, which show 36.9% of treatment failure over the 14-day follow-up period, unadjusted by genotyping. However, with regards to the home treatment of presumed malaria in children (HMM), it has been decided to continue to use pre-packaged chloroquine, either PaluStop^® ^sold by the NGO "Population Service International" (PSI) or Ody Tazomoka^® ^freely distributed at primary public health facilities by Malagasy Ministry of Health (MalMoH), as transitory measure until ACTs were available at community level [8]. At present, the implementation of the ACTs as first-line treatment at health centres level is complete on the east coast of Madagascar, with the result that only 24% (31/131) of the health districts in Madagascar are using ACT.

Since 1999, the antimalarial drug resistance surveillance system in Madagascar is supported by a national network for the surveillance of malaria resistance (named RER – Réseau d'Etude de la Résistance). The RER was formed as a collaborative effort between the MalMoH and the Malaria Research Unit of the Institut Pasteur de Madagascar (IPM). The strategy of monitoring was initially based on the use of the *in vitro *assessment of *P. falciparum *sensitivity to antimalarial drugs and the evaluation of the frequency of genetic markers associated with *P. falciparum *drug resistance [9,10]. *In vivo *studies were also carried out, but these studies were limited geographically, the sample sizes were small and used only a 14-day follow-up period, a series of constraints which may have significantly underestimated the true risk of treatment failure [11-14].

In order to improve the monitoring of antimalarial drug resistance in Madagascar, a new RER was set up in 2006, with the support of Global Fund. According to the WHO recommendation [15], this network was based on sentinel sites located within public or private health facilities and selected to be representative of the range of ecological and epidemiologic conditions. The main purpose of this paper is to report the establishment of the new RER, and to present the up-dated baseline data from multi-site randomized clinical trial assessing the therapeutic efficacy of antimalarial therapies recommended by the National Malaria Control Programme.

## Methods

### Organizational structure and study sites

**Figure 1:**
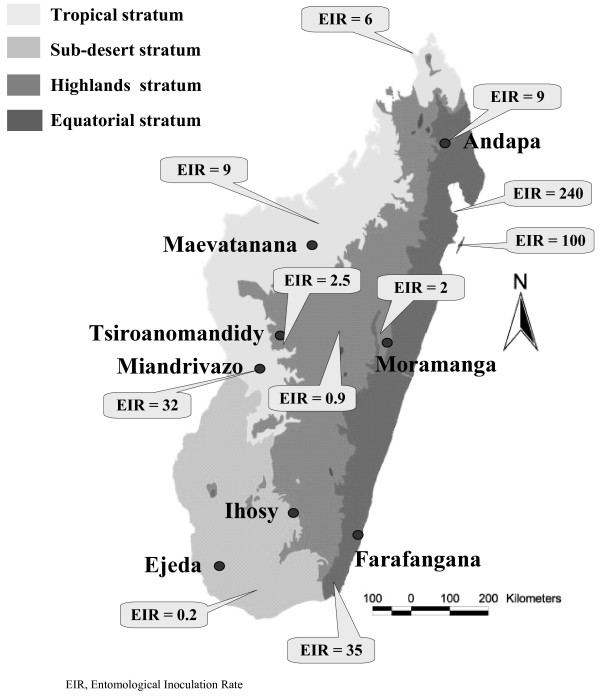
Map of Madagascar based on malarious epidemiological strata and the eight selected sentinel sites involved for monitoring the antimalarial drug resistance [19].

The antimalarial drug efficacy trials were conducted in two steps in eight sentinel sites located in the four different malarious epidemiological strata throughout Madagascar: from February to June 2006, in Ejeda and Ihosy in the South Madagascar (sub-desert stratum, epidemic prone), in Maevatanana and Miandrivazo in the West Madagascar (tropical stratum, seasonal and endemic area) and in Tsiroanomandidy and Moramanga in the foothills of the Central Highlands of Madagascar (highlands stratum, low-endemic area); from March to August 2007 in Andapa and Farafangana in the East Madagascar (equatorial stratum, perennial endemic area) (Figure 1).

### Patient enrolment

This was an open-label trial in which patients with microscopically-confirmed falciparum malaria were randomized into three or four treatment groups according to sentinel sites: CQ, amodiaquine (AQ), SP or ASAQ in Ejeda, Ihosy, Miandrivazo, Maevatanana and Moramanga; CQ, AQ and SP in Tsiroanomandidy and AQ, SP and ASAQ in Andapa and Farafangana.

All patients aged between six months and 15 years were eligible to be enrolled and were screened for malaria at the primary health centres in the sentinel sites on the basis of history of febrile illness. According to the slightly modified WHO 2003 protocol [15], inclusion criteria were (i) monoinfection with *P. falciparum *at a parasitaemia between 1,000 and 200,000/μl, (ii) axillary temperature ≥ 37.5°C, (iii) body weight > 5 kg, (iv) absence of severe malnutrition, (v) absence of febrile conditions caused by diseases other than malaria, (vi) absence of 'danger signs' (inability to stand, breastfeeding or drink; recent convulsions; lethargy or persistent vomiting) and of severe and complicated malaria, (vii) haemoglobin (Hb) ≥ 5 g/dl, and (viii) informed written consent of parents/guardians. Known hypersensitivity to SP, AQ, or AS was considered as an exclusion criterion. Once written informed consent was given, patients were enrolled in the study and assigned consecutive patient numbers. Randomization to treatment group was performed in blocks of three or four, and treatment regimens were allocated by an independent individual, not involved in the analysis of the study.

### Treatment and follow-up procedures

Patients were administered either CQ (10 mg/kg on days 0 and 1, and 5 mg/kg on day 2), AQ (10 mg/kg on days 0, 1, and 2), SP (25 mg/kg sulphadoxine and 1.25 mg/kg pyrimethamine as a single dose on day 0) or ASAQ (AS: 4 mg/kg on days 0, 1, and 2 and AQ:10 mg/kg on days 0, 1, and 2). Patients were directly observed for 30 minutes after treatment, and the dose was readministered if vomiting occurred. Patients who repeatedly vomited their first dose of study medication were excluded from the study.

Patients were assessed on days 1, 2, 3, 7, 14, 21 and 28, and any intervening day they were unwell for malaria infection. Blood was obtained by finger prick on all follow-up days and on any unscheduled day to use for analysis of thick and thin blood smears and for storage on filter paper. Thick and thin blood slides were examined by light microscopy for parasites on any day during the 28-day follow-up. Blood slides were read by a microscopist blind to treatment allocation. All slides were controlled by a second microscopist also blind to treatment group and previous diagnosis. Discordant slides were read, blind to treatment group and previous diagnosis, by a third microscopist. Haemoglobin was measured on Day 0 and Day 28 using a HemoCue haemoglobinometre (HemoCue AB, Ängelholm, Sweden).

### Outcome measures

Treatment outcomes were assessed according to WHO 2003 guidelines as Early Treatment Failure (ETF; danger signs or complicated malaria or failure to adequately respond to therapy on days 0–3), Late Clinical Failure (LCF; danger signs or complicated malaria or fever and parasitemia on days 4–28 without previously meeting criteria for ETF), Late Parasitological Failure (LPF; asymptomatic parasitaemia on days 4–28 without previously meeting criteria for ETF or LCF), and Adequate Clinical and Parasitological Response (ACPR; absence of parasitaemia on day 28 without previously meeting criteria for ETF, LCF, or LPF) [15]. Overall Treatment failure (OFT) was considered as the sum of the ETP, LCT and LPF.

Patients classified as having suffered treatment failure were treated with quinine (10 mg/kg three times daily for seven days); however, their response to repeat therapy was not assessed. Patients were excluded after enrolment if any of the following occurred: (i) use of antimalarial drugs outside of the study protocol; (ii) detection during follow-up of mixed malarial infections (iii) parasitaemia in the presence of a concomitant febrile illness which would interfere with the classification of treatment outcome; (iv) withdrawal of consent; (v) loss to follow-up, (vi) protocol violation, or (vii) death due to a non-malaria illness.

### Laboratory procedures

Blood smears were stained with 10 % Giemsa for 10 min. Parasite densities were determined from thick blood smears by counting the number of asexual parasites per 200 WBCs (or per 500, if the count was less than 10 parasites/200 WBCs), assuming a WBC count of 8,000/μl. A smear was considered negative if no parasites were seen after review of 100 fields. Thin blood smears were used to detect non-falciparum infections [16].

Molecular genotyping techniques were used to distinguish recrudescence from new infection for all patients failing therapy after day 7. Filter paper blood samples collected on the day of enrolment, on day 1 and on the day of failure were analysed for polymorphisms in the genes for merozoite surface protein-1 (*msp-1*) and merozoite surface protein-2 (*msp-2*) using nested-PCR as previously described [17]. First, *msp-2 *genotyping patterns on the day of failure were compared with those at treatment initiation and on day 1, using Quantity One^© ^software (BioRad laboratories, Inc., 1000 Alfred Nobel Drive, Hercules, CA 94547, United States). If all of the *msp-2 *alleles present on the day of failure were present at the time of treatment initiation or on day 1, genotyping was repeated using *msp-1*. An outcome was defined as recrudescence if all *msp-1 *and *msp-2 *alleles present at the time of failure were present at the time of treatment initiation or on day 1, and defined as a new infection otherwise.

### Statistical analysis

Data were entered and verified using EpiInfo 6.04^© ^software (Centers for Disease Control and Prevention, Atlanta, Georgia, United States), and analysed using MedCalc^© ^software version 9.1.0.1 (MedCalc Software, Broekstraat 52, 9030 Mariakerke, Belgium). As the proportion of treatment failures was unknown in the sentinel sites, a minimum of 50 patients was included for each treatment group and each site, according to available human and financial resources and the WHO recommendations [15].

Efficacy data were assessed with a per-protocol analysis that included all patients who completed the study. An age-stratified analysis for patients less than five years of age and patients five years or older was planned. This study was not designed for the primary analysis to be stratified by site. Parasite densities were normalized using logarithmic transformation. Categorical variables were compared using χ^2 ^or Fisher's exact test, and continuous variables were compared using an independent samples *t *test.

The primary efficacy outcomes were 14-day and 28-day clinical and parasitological failure risks both unadjusted and adjusted by genotyping. Risks of clinical and parasitological treatment failure after adjustment by genotyping were estimated using Kaplan-Meier survival analysis techniques in accordance with the new WHO protocol [15]. With survival analysis, data were censored for new infections. Secondary outcomes included fever clearance, parasite clearance, change in haemoglobin levels between Day 0 and the last day of follow-up, and the incidence of adverse events. Hypothesis testing was made using risk differences, exact 95% confidence intervals, and *P *values. A *P *value (two-tailed) of less than 0.05 was considered statistically significant.

### Ethical approval

The study protocol was reviewed and approved by the Ethics Committee of the Ministry of Health of Madagascar (N°007/SANPF/2007). An informed written consent was provided by the parents/guardians of all patients before they were included in the study.

## Results

### Enrolment

**Figure 2:**
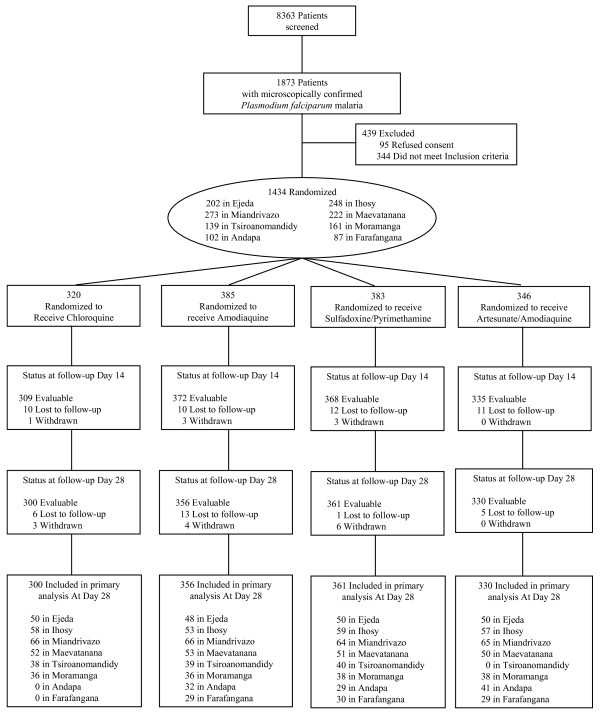
Flow of patients.

In the eight sentinel sites, 8,363 patients were screened, with 1,873 patients positive for *P. falciparum *(22.4%). Of these patients, a total of 1,434 patients were enrolled in February-June 2006 and March-July 2007 (76.6%). A total of 320 patients were randomized to CQ, 385 to AQ, 383 to SP, and 346 to ASAQ. The main reasons for patients with *P. falciparum *not being included were non-consent, inability to attend follow-up or concurrent disease. The flow of patients through the trial is shown in Figure 2.

A total of 1,347 of 1,434 patients (93.9%) completed treatment and follow-up to day 28 (92.5% in the AQ group, 93.7% in the CQ group, 94.2% in the SP group, and 95.4% in the ASAQ group). Eighty eight patients were either lost to follow-up (n = 68) or excluded/withdrawn from the trial (n = 20). Of these patients, 18 withdrew consent, two were withdrawn due to adverse events. The baseline characteristics of patients across the treatment groups were similar at each site (Table 1).

**Table 1:** Baseline characteristics by treatment group*

	Treatment Group			
	
Characteristics	Chloroquine	Amodiaquine	Sulphadoxine-Pyrimethamine	Artesunate-Amodiaquine
No. of patients enrolled	320	385	383	346
Ejeda	51	51	50	50
Ihosy	62	62	61	63
Miandrivazo	67	69	69	68
Maevatanana	55	56	56	55
Tsiroanomandidy	43	48	48	0
Moramanga	42	39	40	40
Andapa	0	32	29	41
Farafangana	0	28	30	29
Age				
median (range), y	5 (0.5 – 15)	5 (0.5 – 15)	5 (0.5 – 15)	5 (0.5 – 15)
Proportion of children ≤ 5 years	142 (45.4)	185 (48.7)	182 (47.8)	167 (48.7)
Weight, median (IQR), kg	15 (6 – 58)	15 (5 – 62)	14 (6 – 60)	13 (2.6 – 56)
Female	158 (49.5)	188 (48.8)	186 (48.6)	169 (49.0)
Temperature, mean (95% CI)	38.4 (38.3 – 38.5)	38.4 (38.3 – 38.5)	38.4 (38.3 – 38.5)	38.6 (38.5 – 38.7)
Trophozoite density, geometric mean (range), parasite/μl	15,302 (1,241 – 200,000)	133,068 (1,000 – 194,000)	13,880 (1,000 – 196,000)	14,346 (1669 – 192000)
Haemoglobin, median (IQR), g/dL	10.1 (5.3 – 15.8)	10.2 (5.8 – 15.6)	10.0 (5.6 – 14.7)	10.1 (5.1 – 14.5)
Anemic at enrollment^±^	107 (39.3)	114 (34.5)	127 (38.5)	103 (35.4)

Abbreviations: CI, confidence interval; IQR, interquartile range.

*Data are presented as No. (%) unless otherwise indicated.

†Anaemia defined as haemoglobin of less than 10 g/dL.

### Primary outcome: treatment efficacy

**Figure 3:**
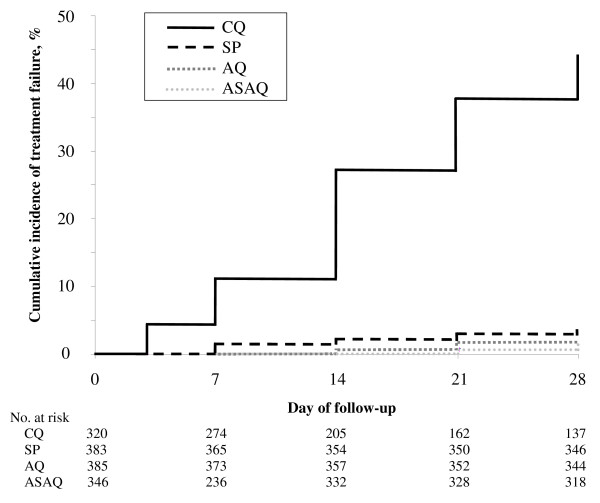
Kaplan-Meier curve of cumulative treatment failure over the 28-day follow-up period, adjusted by genotyping.

The results of the treatment efficacy are presented by treatment group and day of follow-up in Table 2 and by age group and study site in Tables 3 and 4. Figure 3 showed the Kaplan-Meier curve of cumulative treatment failure over the 28-day follow-up period, adjusted by genotyping.

**Table 2:** Treatment outcomes stratified by treatment group and day of follow-up

**Outcome Measure by Day**		**No./Total (%) of Patients [95% CI]**											
		
		**CQ**			**SP**			**AQ**			**ASAQ**		
***Day 14***													
Clinical failure	ETF	14/309	(4.5)	[2.6–7.3]	4/368	(1.1)	[0.3–2.6]	2/372	(0.5)	[0.1–1.7]	0/335	(0)	
	LCF	29/309	(9.4)	[6.5–13.0]	2/368	(0.5)	[0.1–1.7]	0/372	(0)		0/335	(0)	
Parasitological failure	LPF	56/309	(18.1)	[14.1–22.7]	3/368	(0.8)	[0.2–2.2]	0/372	(0)		0/335	(0)	
Overall Treatment failure		99/309	(32.0)	[27.0–37.4]	9/368	(2.4)	[1.2–4.4]	2/372	(0.5)	[0.1–1.7]	0/335	(0)	

***Day 28 Unadjusted by genotyping***													
Clinical failure	ETF	14/300	(4.7)	[2.7–7.5]	4/361	(1.1)	[0.4–2.7]	2/356	(0.6)	[0.1–1.8]	0/330	(0)	
	LCF	45/300	(15.0)	[11.3–19.4]	4/361	(1.1)	[0.4–2.7]	3/356	(0.8)	[0.2–2.3]	4/330	(1.2)	[0.4–2.9]
Parasitological failure	LPF	104/300	(34.7)	[29.4–40.2]	8/361	(2.2)	[1.0–4.2]	6/356	(1.7)	[0.7–3.5]	8/330	(2.4)	[1.1–4.6]
Overall Treatment failure		163/300	(54.4)	[48.7–59.9]	16/361	(4.4)	[2.6–6.9]	11/356	(3.1)	[1.6–5.3]	12/330	(3.6)	[2.0–6.1]

***Day 28 Adjusted by genotyping***													
Clinical failure	ETF	14/300	(4.7)	[2.7–7.5]	4/361	(1.1)	[0.4–2.7]	2/356	(0.6)	[0.1–1.8]	0/330	0	[0.4–2.7]
	LCF	34/300	(11.3)	[8.1–15.3]	3/361	(0.8)	[0.2–2.2]	1/356	(0.3)	[0–1.4]	2/330	(0.6)	[0.1–2.0]
Parasitological failure	LPF	84/300	(28.0)	[23.1–33.3]	5/361	(1.4)	[0.5–3.0]	3/356	(0.8)	[0.2–2.3]	4/330	(1.2)	[0.4–2.9]
Overall Treatment failure		132/300	(44.0)	[38.5–49.7]	12/361	(3.3)	[1.8–5.6]	6/356	(1.7)	[0.7–3.5]	6/330	(1.8)	[0.7–3.7]

CI: Confidence Interval; ETF: Early Treatment Failure; LCF: Late Clinical Failure; LPF: Late Parasitological Failure; CQ: Chloroquine; AQ: Amodiaquine; ASAQ: Artesunate + Amodiaquine; SP: Sulphadoxine-Pyrimethamine

**Table 3:** Treatment failures by Day 28 (adjusted by genotyping), stratified by age group

**Treatment groups**	**No. of treatment failure/Total of Patients (%) [95% CI]**		
	
	**Age groups**		Significance level P*
	
	**0.5 – 4 years**	**≥ 5 years**	
**CQ**	69/131 (52.6) [44.1–61.1]	63/169 (37.3) [30.2–44.7]	0.01
**SP**	8/171 (4.7) [2.2–8.7]	3/190 (1.6) [0.4–4.2]	0.57
**AQ**	4/174 (2.3) [0.7–5.5]	2/182 (1.1) [0.2–3.6]	0.64
**ASAQ**	4/161 (2.8) [0.8–5.9]	2/169 (1.2) [0.2–3.9]	0.51

CQ: Chloroquine; AQ: Amodiaquine; ASAQ: Artesunate + Amodiaquine; SP: Sulphadoxine-Pyrimethamine.

CI: Confidence Interval

Chi-square test for the comparison of two proportions

**Table 4:** CQ Treatment failures by Day 28 (adjusted by genotyping), stratified by age group and study site

**CQ**		**No. of treatment failure/Total of Patients (%) [95% CI]**		
		
		**0.5 – 4 years**	**≥ 5 years**	**All age**
**Tropical**	**Maevatanana**	19/28 (67.9) [49.1–83.0]	9/24 (37.5) [20.1–57.8]	28/52 (53.8) [40.3–67.0]
	**Miandrivazo**	10/16 (62.5) [37.6–83.2]	16/50 (32.0) [20.2–45.8]	26/66 (39.4) [28.2–51.5]
**Highlands**	**Moramanga**	10/26 (38.5) [21.5–57.9]	3/10 (30.0) [8.3–62.0]	13/36 (36.1) [21.8–52.6]
	**Tsiroanomandidy**	13/21 (61.9) [40.2–80.5]	9/17 (52.9) [29.7–75.2]	22/38 (57.9) [41.9–72.7]
**Sub-desert**	**Ejeda**	14/20 (70.0) [47.7–86.8]	18/30 (60.0) [41.9–76.2]	32/50 (64.0) [50.1–76.4]
	**Ihosy**	3/20 (15.0) [4.0–35.6]	8/38 (21.1) [10.3–36.1]	11/58 (19.0) [10.4–30.6]

CQ: Chloroquine. CI: Confidence Interval. Chi-square test for the comparison of two proportions

All treatment regimens, except for the CQ treatment group, resulted in clinical cure rates above 97.6% by day-14 and 96.7% by day-28 (adjusted by genotyping). Considering all sites, the OTF of the CQ group was significantly higher than in the other treatment group as well as day 14 or day 28 unadjusted or adjusted by genotyping (*P *< 0.0001).

In the CQ group, ETF was uncommon with only 10.6% of the OTF (i.e., 14/132). Most of the treatment failures adjusted by genotyping were occurred in the second/third week of follow-up mainly as LPF (63.6%). Among the eight sites, the OTF adjusted by genotyping of CQ was significantly different ranging from 19% in Ihosy to 64% in Ejeda (*P *< 0.0001). The risk of treatment failure adjusted by genotyping in children less than five years old was almost twice as frequent than in older patients (52.7% *vs*. 37.3%, adjusted odds ratio [OR], 1.8; 95% confidence interval [CI], 1.2–3.0; *P *= 0.004). The risk of treatment failure by day-14 was significantly lower than by day-28 adjusted by genotyping (32.0% *vs*. 44.0%, risk difference 12.0%, 95% CI: 4.3%–19.5%, *P *= 0.003).

In the SP group, treatment failures were rare with an OTF adjusted by genotyping of 3.3%, but ETF was accounting for more than one third of the treatment failures: Two patients from Ejeda developed danger signs despite decreasing parasite density, one patient from Tsiroanomandidy presented at day 3, a parasite density > 25% of count on day 0 and the last patient from Maevatanana, a parasite density with an axillary temperature ≥ 37.5°C. The distribution of the treatment failure adjusted by genotyping was not significantly different among the sites (*P *= 0.08), especially because of the small number of the patients included per site and the small number of treatment failure.

In the AQ and ASAQ groups, treatment efficacies were globally very high throughout Madagascar. Treatment failures adjusted by genotyping were not significantly different among the sentinel sites (*P *= 0.24 for AQ and *P *= 0.12 for ASAQ).

### Secondary outcomes

**Figure 4:**
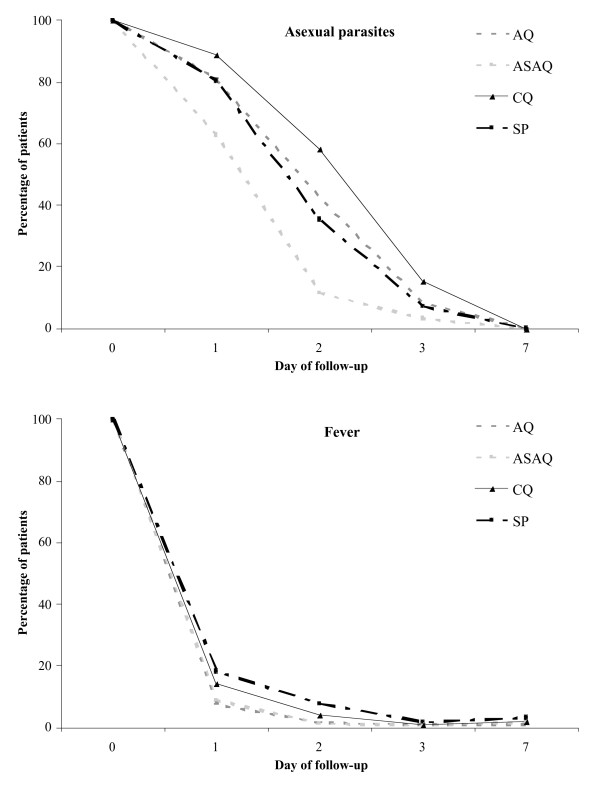
Proportions of patients with asexual parasites and fever (T° ≥ 37.5°C) on days of follow-up by treatment group.

Parasite clearance was more rapid with ASAQ than CQ, AQ or SP until day 3 (P < 0.0001). Fever clearance was delayed with CQ and SP, the proportion of febrile patients being significantly lower with ASAQ and AQ until day-2. The details are shown on Figure 4. On day-28, the extent of haematological recovery (median of individual increases in Hb) did not differ significantly between the four groups (CQ, 0.8 g/dl, -4.1 to 4.9; SP, 1.0 g/dl, -4.8 to 6.4; AQ, 1.0 g/dl, -4.4 to 8.3; ASAQ, 0.9 g/dl, -6.0 to 6.4).

No severe side-effects attributable to the study medication were observed during the follow-up period. Minor side effects as vomiting were reported between day 1 and day 3 in seventeen patients (1.26%): 8 in the AQ group, 4 in the CQ and the SP groups and 1 in the ASAQ group.

## Discussion

As effective case management remains the cornerstone of malaria elimination [18], the monitoring of antimalarial drugs resistance is essential to ensure a continued coverage of effective antimalarial treatment. With the support of resources from the Global Fund, the main objective of the new RER was to set up an expanded programme to monitor antimalarial drug resistance in Madagascar, based on sentinel sites. The selected sentinel sites were chosen at peripheral level (within public or private health facilities) to be representative of the four epidemiological strata in which the country could divided [19]. Because of the size of the country and the availability of financial and human resources, two sites were selected by epidemiological stratum for establishing a surveillance system for *in vivo *drug efficacy. Seven additional sites were secondly incorporated in the RER for monitoring *in vitro *drug resistance (*in vitro *drug sensitivity assay and molecular markers) to provide complementary data and warning signals of the possible emergence of resistance or trends of declining drug efficacy.

The objective of the new RER was firstly to establish up-dated baseline data on the efficacy of the antimalarial drugs recommended by the MalMoH in its new Madagascar national policy. The methodology used was based on the WHO 2003 protocol [15], with some modifications (compromise between the high transmission and the low to moderate transmission protocols): (i) children enrolled in the study were aged between six months and 15 years and (ii) of *P. falciparum *parasitaemia on inclusion was between 1,000 and 200,000/μl. The protocol included randomized, 28-day follow-up with genotyping to discriminate recrudescence from new infections, and assessment of safety and tolerability. Despite the complexity of conducting a multi-site study in Madagascar, an excellent completion rates was achieved (> 93%) and high-quality data were collected.

The four main antimalarial drugs used and recommended by the NMCP were evaluated in five sites. In Tsiroanomandidy, ASAQ was not evaluated because this drug was already assessed in another clinical trial [20] and in Andapa and Farafangana, CQ was not evaluated for ethical reasons because of the too high frequency of treatment failure found in the previous clinical trial in 2006.

Two methodological points could be underline by the results of the study: (1) the necessity to extend the follow-up to a minimum of 28 days (even more for drugs with a prolonged duration of action) to avoid the underestimation of the risk of treatment failure [21], i.e. for CQ, most of the treatment failures occurred between 15 and 28 days after treatment, a 14 day follow-up will underestimated the OTF of 12% (32.0% for 14-days instead of 44.0% for 28-days) and (2) the necessity to enrol sufficient number of patients to allow the stratification of the result based on age (< 5 years and ≥ 5 years), i.e. the risk of CQ treatment failure in children < 5 years was 2-fold more frequent than in older patients (five to 15 years old), especially in moderate to high transmission areas (Maevatanana and Miandrivazo).

This multi-site study represents the first extent *in vivo *drug efficacy study performed in Madagascar since 1983 [22-24]. The CQ efficacy was signifantly different between the eight sites and ranged from moderate (Ihosy, 81%), intermediate (Miandrivazo, 60.6% and Moramanga, 63.9%) to low (Maevatanana, 46.2%; Tsiroanomandidy, 42.1% and Ejeda, 36%), showing the important of the multi-site studies. However, the results about the CQ efficacy were comparable to those found previously in the last in vivo trial carried out in Sainte Marie on the east coast in 2004 (36.9% of clinical failure within two weeks of CQ treatment). Currently, CQ remains the drug most widely available (distribution and financial criteria) and is the first drug used in most of areas in Madagascar at community level, for treating acute fever before or without laboratory diagnosis. According to Hastings *et al *[6], even with 50% of efficacy, CQ might still be perceived as very effective at community level, especially because of its antipyretic activity that alleviate symptoms and because that most of the recrudescence's which occur two to three week later after the treatment are not perceived as a failure. Based on these findings, the MalMoH must improve the HMM, as soon as possible, by withdrawing pre-packaged CQ and gradually replacing it with pre-packaged ASAQ.

The results concerning the SP efficacy were more informative, contrasting with the good effectiveness reported in previous studies in 2003–2004 [13,14]. The more worrying was to finding even if the treatment failures were rare that the ETFs were accounting for more than one third of the SP treatment failure. There is no doubt that with the increased drug use of SP in the next few years in Madagascar as IPT for pregnant women, the monitoring of its drug resistance especially with the useful and reliable molecular makers such as *Pf-dhfr *and *Pf-dhps *[25] will be make it a priority for the RER.

This study also confirmed that ASAQ, the first-line treatment of the uncomplicated malaria recommended in Madagascar was very effective, particularly because of the excellent effectiveness of AQ, the partner drug associated with artesunate. Although it was not feasible to monitor for the potentially serious adverse effects associated with amodiaquine such as neutropenia or hepatotoxicity (the sample size was too small to exclude any clinically toxicity with a low frequency such as < 1:100), ASAQ was well tolerated in the study population. This combination produced also faster parasite and fever clearances than the other antimalarial treatments. These findings constitute important baseline data on the efficacy of this combination, a key tool within the framework of the elimination of the malaria [18].

The results from this multi-site study show that antimalarial drug resistance remains low in Madagascar, except for CQ, compared to the bordering countries in the Indian Ocean region such as the Comoros Archipelago [26,27]. The next step must be to complete these data by providing the frequency of mutant parasites correlated with the CQ-resistance (*Pfcrt*) or SP-resistance (*Pf-dhfr *or *Pf-dhps*) or the *in vitro *response of parasites for drug for which no validate molecular marker are available such as amodiaquine, artemisinin derivatives or amino alcohols (quinine, lumefantrine). These complementary indicators should allow the early detection of the spread of *P. falciparum *drug resistance, especially those introduced from the Comoros Islands [28]. Indeed, the extension of the RER at regional level is also crucial to enable the countries of the region to share national information on antimalarial drug efficacy and to help to minimize delays in implementing national antimalarial drug policy change.

## Authors' contributions

DM contributed to the design and coordination of the study, supervised the enrolment and follow up of the patients, assisted with data entry and interpretation and prepared the manuscript. ARat supervised the enrolment and follow up of the patients. LR, ARan and RR performed field work in Tsiroanomandidy and Moramanga. M-AR and ER were involved in laboratory work. MJ and VA carried out molecular genotyping. MR, L-PR, LR, OD, LT and ARav helped to compose the manuscript and gave constructive advice.
